# Knowledge, attitude and practice of patients towards orthodontic treatment

**DOI:** 10.1186/s12903-023-02780-y

**Published:** 2023-03-08

**Authors:** Roselyn Mathew, Hans Prakash Sathasivam, Lisa Mohamednor, Prethiba Yugaraj

**Affiliations:** 1 Bangsar Dental Clinic, Ministry of Health, Jalan Bangsar, 59200 Federal Territory of Kuala Lumpur, Malaysia; 2 Institute for Medical Research, National Institutes of Health, Ministry of Health, 40170 Shah Alam, Selangor Malaysia; 3 Clinical Research Centre, Hospital Putrajaya, Ministry of Health, Jalan P9, Presint 7, 62250 Putrajaya, Federal Territory of Putrajaya, Malaysia

**Keywords:** Orthodontics, Knowledge, Attitude, Practice, Health behaviour

## Abstract

**Background:**

Orthodontic treatment is a time-consuming and highly technique-sensitive clinical procedure. A patient's comprehension and compliance with oral hygiene instructions and appliance maintenance are critical to the success of orthodontic treatment. This study was performed to assess the knowledge, attitude and practice of patients seen at government orthodontic clinics in the Federal Territories of Kuala Lumpur and Putrajaya towards orthodontic treatment.

**Methods:**

A validated, bilingual, self-administered questionnaire comprising fifteen questions across the domains of Knowledge, Attitude and Practice was used and responses were assessed with 3 responses; one correct, one incorrect and one reflecting uncertainty. 507 patients from five orthodontic centres participated in this study. Data was analysed using SPSS. Continuous data was summarised as mean and standard deviation or median and inter-quartile range, as appropriate. Categorical data was summarised as frequency and percentage, then univariable analysis was carried out with Pearson’s chi-square test or Fisher’s exact test, as appropriate.

**Results:**

The mean age of respondents was 22.5 years (SD ± 2.8). A majority of respondents were female (64.1%) and from the lowest income bracket or B40 group (71%). Overall, for the knowledge domain, a majority of the respondents got all questions correct. 69.4% of patients were aware that incomplete treatment could lead to worsening of their malocclusion. 80.9% of respondents were aware of the need for a retainer upon completion of their orthodontic treatment. For the attitude section, 64.7% felt that they had to wait a very long time to see the orthodontist. In the Practice domain, the majority only got two of the five questions correct. Only 39.8% of respondents made an effort to alter dietary habits all of the time. In general, females and those with tertiary education fared better for all three domains.

**Conclusions:**

The orthodontic patients in the Federal Territories of Kuala Lumpur and Putrajaya possess good knowledge about their treatment however their attitude and orthodontic related practices need to be improved.

## Background

Malocclusion is a relatively common oral health problem that can adversely affect an individual’s quality of life as it influences not only oro-facial functions such as mastication, speech and swallowing, but also exerts some amount of psycho-social effect due to its impact on aesthetics [1]. A recent systematic review and meta-analysis on the prevalence of malocclusion in children and adolescents concluded that the worldwide prevalence was around 56% [2]. In Malaysia, it has been found that around 30% of children are in need of orthodontic treatment [3]. Patients usually seek orthodontic treatment to improve either their aesthetics or oral function such as masticatory efficiency [4–6].

The success of orthodontic treatment depends on a multitude of factors related to patient compliance, such as attending appointments, maintaining good oral hygiene and limiting breakages of appliance [7, 8]. Patients who have had previous orthodontic treatment and parents who themselves have had orthodontic treatment tend to have better attitude towards orthodontic treatment [9]. Studies have illustrated that patients with better knowledge of orthodontics have improved attitudes towards orthodontic treatment thus increasing the likelihood of improved clinical outcome [10, 11].

The responsibility of assessing patient compliance and imparting the necessary knowledge to patients before and throughout the entire course of the orthodontic treatment usually falls on the shoulder of the orthodontist and orthodontic team. Having prior information on the knowledge, attitude and practice (KAP) of patients can be extremely useful in aiding the orthodontist in not only estimating a patient’s compliance level but also in formulating strategies to improve patient compliance which may in turn improve clinical outcomes.

Though such studies have been conducted in other parts of the world to assess the level of knowledge, attitude and practice in orthodontic patients, they are few and far between [12–14]. Most of these studies found that the majority of orthodontic patients possess good knowledge of orthodontic treatment but were lacking with regard to attitude and practice. There is a lack of such studies in the Malaysian setting. As such, the aim of our study was to evaluate knowledge, attitude and practices of the patient towards orthodontic treatment in a Malaysian population and to compare and contrast our findings with those from the rest of the world.

## Methods

### Patients

Only patients who were literate in either English or Malay were selected. Informed consent was obtained from all patients prior to recruitment. Consecutive patients were recruited from five public orthodontic clinics over a period of six months (April to September 2021). Patients aged 18-years old and above who have been undergoing orthodontic treatment using fixed-appliance for a minimum period of six months were included in the study. Patients with any of the following characteristics were excluded: (1) patients with medical problems such as psychological disorders and learning disabilities: (2) patients with dento-facial deformities (such as cleft palate): (3) patients undergoing / undergone non-fixed appliance.

### Questionnaire

The questionnaire was adapted from a previously published study by Shrestha et al. [13]. Permission to use the questionnaire was obtained from the corresponding author of the aforementioned study. The questionnaire was modified to better suit the local setting. Three dental specialists assessed the modified version for content validity. The questionnaire was then, translated into the Malay language by two language experts and the best version of the questionnaire was then constructed. Backward translation was performed by another two language experts blinded to the original questionnaire. The backward translation was found to be very close to the original modified questionnaire. Three dental specialists then content validated the translated version. Item-level content validity index (I-CVI) was 0.82 and scale-level content validity index S-CVI/UA was 0.8. Face validity of the questionnaire was then tested on 20 subjects and scale-level face validity-index based on average method (S-FVI/Ave) was 0.99 and scale-level face validity index based on the universal agreement method (S_FVI/UA) was 0.93. Reliability of the questionnaire was evaluated and the 15-item questionnaire had a Cronbach’s alpha value of 0.702, indicating an acceptable level of reliability. From this, the final self-administered questionnaire was prepared in both English and Malay languages. Google Forms was selected to be the platform to be used for the questionnaire.

The questionnaire comprised four sections, the first being socio-demographic variables of study participants which include age, sex, race, education level and household income. The 2nd, 3rd and 4th sections were on knowledge, attitude and practice respectively with 5 questions per section with three possible responses; one correct, one incorrect and one reflecting uncertainty. The level of education was reclassified as those with tertiary education and those with lower than tertiary education. Household income was categorized into three categories based on the Malaysian Department of Statistics’ (DOSM) Household Income and Basic Amenities (HIS/BA) survey of 2019: (1) Top 20% (T20): (2) Middle 40% (M40): (3) Bottom 40% (B40) [15]. The income level was then regrouped into the bottom 40% B40 and non-B40 group.

The data obtained were then regrouped so that the positively formulated knowledge was deemed as correct and negatively formulated knowledge and those uncertain were deemed as incorrect. As for attitude and practice the positive formulated attitudes and practices that are consistent with the current orthodontic recommendation regrouped as correct and those uncertain and the negatively formulated attitudes and practices were regrouped as incorrect.

### Statistical analysis

Descriptive statistical analysis was performed using IBM SPSS Statistics for Windows version 21 (IBM Corp., Armonk, N.Y., USA). Continuous data was summarised as mean and standard deviation or median and inter-quartile range, as appropriate. Categorical data was summarised as frequency and percentage, then univariable analysis was carried out with Pearson’s chi-square test or Fisher’s exact test, as appropriate. Results were considered statistically significant at *p* < 0.05 value unless stated otherwise.

## Results

A total of 507 respondents with a mean age of 22.5 years (SD ± 2.8) participated in this study. The socio-demographic features of the respondents are shown in Table 1. Briefly, participants were predominantly female (64.1%) and majority of respondents were from the Malay ethnic group (62.3%). Almost two-thirds of respondents (74.3%) had tertiary education. Most respondents were from the lowest income bracket or B40 group (71%), followed by 23.7% of respondents from the middle-income bracket (M40 group) and only 5.3% of respondents were from the highest income group (T20 group).

**Table 1:** Demographic details of study participants

Characteristic	n (%)
Age in years, Mean [SD]	22.5 [2.8]
Sex	
Male	182 (35.9)
Female	325 (64.1)
Race	
Malay	316 (62.3)
Chinese	137 (27)
Indian	47 (9.3)
Others	7 (1.4)
Education level	
No formal education	8 (1.6)
Primary	1 (0.2)
Secondary	121 (23.9)
Tertiary	377 (74.3)
Household income	
B40 (< RM2500–RM 4849)	360 (71.0)
M40 (RM4850–RM 10,959)	120 (23.7)
T20 (10,960 and above)	27 (5.3)

*SD* standard deviation

### Knowledge (K) of orthodontic treatment

For all five of the knowledge questions, a vast majority of the respondents provided the “correct” response. The vast majority of respondents, 97.8% knew the purpose of braces (Table 2; K1). A large majority, 86.6% knew that the treatment duration was long (Table 2; K2) and 98.0% knew of the importance of following the oral hygiene instructions as well as dietary restrictions (Table 2; K3). Interestingly, only 69.4% of patients were aware that incomplete treatment could lead to worsening of their malocclusion over time (Table 2; K4). 80.9% of respondents were aware of the need for a retainer after completion of orthodontic treatment (Table 2; K5). Regarding the need for retainers, females (*p* = 0.017) (Table 2; K5) and those with tertiary level of education (*p* = 0.008) (Table 3; K5) had significantly better knowledge. Respondents with tertiary education also had significantly better knowledge about the duration of their orthodontic treatment (*p* = 0.011) (Table 3; K2) and on the need of retainers when compared to other levels of education (*p* = 0.008) (Table 3; K5).

**Table 2:** Responses of study participants based on sex

Questions	Total (N = 507)		Males		Females		*p* value
	n	%	n	%	n	%	
K1) Braces							
(i) Correct	496	97.8	176	96.7	320	98.5	0.192**
(ii) Incorrect	11	2.2	6	3.3	5	1.5	
K2) The duration of undergoing orthodontic treatment is							
(i) Correct	439	86.6	156	85.7	283	87.1	0.666**
(ii) Incorrect	68	13.4	26	14.3	42	12.9	
K3) Instructions on oral hygiene and eating habits							
(i) Correct	497	98	176	96.7	321	98.8	0.109**
(ii) Incorrect	10	2	6	3.3	4	1.2	
K4) If the treatment is incomplete, the condition of my teeth will							
(i) Correct	352	69.4	123	67.6	229	70.5	0.500**
(ii) Incorrect	155	30.6	59	32.4	96	29.5	
K5) After my braces are removed							
(i) Correct	410	80.9	137	75.3	273	84	0.017**
(ii) Incorrect	97	19.1	45	24.7	52	16	
A1) I think that people who wear braces do not look attractive							
(i) Correct	373	73.6	117	64.3	256	78.8	0.000**
(ii) Incorrect	134	26.4	65	35.7	69	21.2	
A2) I often wait a very long time in the waiting area							
(i) Correct	179	35.3	59	32.4	120	36.9	0.309**
(ii) Incorrect	328	64.7	123	67.6	205	63.1	
A3) Time spent with the orthodontist during my appointment is							
(i) Correct	420	82.8	154	84.6	266	81.8	0.428**
(ii) Incorrect	87	17.2	28	15.4	59	18.2	
A4) I am happy with the treatment outcome so far							
(i) Correct	404	79.7	145	79.7	259	79.7	0.995**
(ii) Incorrect	103	20.3	37	20.3	66	20.3	
A5) My orthodontic treatment is expensive							
(i) Correct	378	74.6	123	67.6	255	78.5	0.007**
(ii) Incorrect	129	25.4	59	32.4	70	21.5	
P1) Restrict certain food and alter my dietary habits to maintain braces							
(i) Correct	202	39.8	73	40.1	129	39.7	0.927**
(ii) Incorrect	305	60.2	109	59.9	196	60.3	
P2) Brush and rinse mouth more often so that food does not get stuck in the braces							
(i) Correct	391	77.1	132	72.5	259	79.7	0.065**
(ii) Incorrect	116	22.9	50	27.5	66	20.3	
P3) While undergoing orthodontic treatment, I clean my teeth using							
(i) Correct	295	58.2	91	50	204	62.8	0.005**
(ii) Incorrect	212	41.8	91	50	121	37.2	
P4) Brackets/wires often break or dislodge due to my carelessness							
(i) Correct	260	51.3	85	46.7	175	53.8	0.123**
(ii) Incorrect	247	48.7	97	53.3	150	46.2	
P5) Often forget or miss my appointment dates							
(i) Correct	397	78.3	125	68.7	272	83.7	0.000**
(ii) Incorrect	110	21.7	57	31.3	53	16.3	

**Pearson’s Chi Square test

Bold entries highlight the statistically significant value

**Table 3:** Responses of study participants based on education level

Questions	Total (N = 507)		Less than tertiary		Tertiary		*p* value
	n	%	n	%	n	%	
K1) Braces							
(i) Correct	496	97.8	105	94.8	391	98.7	0.008**
(ii) Incorrect	11	2.2	6	5.4	5	1.3	
K2) The duration of undergoing orthodontic treatment is							
(i) Correct	439	86.6	88	79.3	351	88.6	0.011**
(ii) Incorrect	68	13.4	23	20.7	45	11.4	
K3) Instructions on oral hygiene and eating habits							
(i) Correct	497	98.0	108	97.3	389	98.2	0.531**
(ii) Incorrect	10	2.0	3	2.7	7	1.8	
K4) If the treatment is incomplete, the condition of my teeth will							
(i) Correct	352	69.4	75	67.6	277	69.9	0.630**
(ii) Incorrect	155	30.6	36	32.4	119	30.1	
K5) After my braces are removed							
(i) Correct	410	80.9	80	72.1	330	83.3	0.008**
(ii) Incorrect	97	19.1	31	27.9	66	16.7	
A1) I think that people who wear braces do not look attractive							
(i) Correct	373	73.6	66	59.5	307	77.5	0.000**
(ii) Incorrect	134	26.4	45	40.5	89	22.3	
A2) I often wait a very long time in the waiting area							
(i) Correct	179	35.3	38	34.2	141	35.6	0.789**
(ii) Incorrect	328	64.7	73	65.8	255	64.4	
A3) Time spent with the orthodontist during my appointment is							
(i) Correct	420	82.8	88	79.3	332	83.8	0.260**
(ii) Incorrect	87	17.2	23	20.7	64	16.2	
A4) I am happy with the treatment outcome so far							
(i) Correct	404	79.7	91	82.0	313	79.0	0.496**
(ii) Incorrect	103	20.3	20	18.0	83	21.0	
A5) My orthodontic treatment is expensive							
(i) Correct	378	74.6	77	69.4	301	76.0	0.156**
(ii) Incorrect	129	25.4	34	30.6	95	24.0	
P1) Restrict certain food and alter my dietary habits to maintain braces							
(i) Correct	202	39.8	47	42.3	155	39.1	0.543**
(ii) Incorrect	305	60.2	64	57.7	241	60.9	
P2) Brush and rinse mouth more often so that food does not get stuck in the braces							
(i) Correct	391	77.1	86	77.5	305	77.0	0.919**
(ii) Incorrect	116	22.9	25	22.5	91	23.0	
P3) While undergoing orthodontic treatment, I clean my teeth using _							
(i) Correct	295	58.2	54	48.6	241	60.9	0.021**
(ii) Incorrect	212	41.8	57	51.4	155	39.1	
P4) Brackets/wires often break or dislodge due to my carelessness							
(i) Correct	260	51.3	55	49.5	205	51.8	0.679**
(ii) Incorrect	247	48.7	56	50.5	191	48.2	
P5) Often forget or miss my appointment dates							
(i) Correct	397	78.3	87	78.4	310	78.3	0.983**
(ii) Incorrect	110	21.7	24	21.6	86	21.7	

**Pearson’s Chi Square test

Bold entries highlight the statistically significant value

### Attitude (A) towards orthodontic treatment

Generally, the respondents had a good or positive attitude towards orthodontic treatment with a vast majority of respondents getting four out of the five attitude-related questions correct. A majority of the respondents (64.7%) felt that they had to wait a very long time to see the orthodontist (Table 2; A2). Almost three-quarters of the respondents (73.6%) had a positive attitude on the attractiveness of patients wearing braces (Table 2; A1). The remaining 26.4% were either undecided on the attractiveness or felt that they did not look good with braces. 82.8% felt that the time spent with the orthodontist during follow-up visits was sufficient (Table 2; A3). The vast majority of respondents (79.7%) felt satisfied with the treatment outcome thus far and 74.6% felt that the cost of the orthodontic treatment provided in the government clinic was inexpensive (Table 2; A4 and A5). Females and those with tertiary education (Table 2; A1 and Table 3; A1) were found to have a more positive attitude on the attractiveness of patients who had braces and this finding was statistically significant (*p* < 0.001). Female patients felt that the government orthodontic service was affordable and this difference between the sexes was statistically significant (*p* = 0.007) (Table 2; A5). For the same question there were also a significantly higher number of patients from the non-B40 group who felt that orthodontic treatment at government clinics was affordable (*p* = 0.010) (Table 4; A5).

**Table 4:** Responses of study participants based on income level

Questions	Total (N = 507)		B40		NON-B40		*p* value
	n	%	n	%	n	%	
K1) Braces							
(i) Correct	496	97.8	351	97.5	145	98.6	0.424**
(ii) Incorrect	11	2.2	9	2.5	2	1.4	
K2) The duration of undergoing orthodontic treatment is							
(i) Correct	439	86.6	312	86.7	127	86.4	0.935**
(ii) Incorrect	68	13.4	48	13.3	20	13.6	
K3) Instructions on oral hygiene and eating habits							
(i) Correct	497	98	354	98.3	143	97.3	0.438**
(ii) Incorrect	10	2	6	1.7	4	2.7	
K4) If the treatment is incomplete, the condition of my teeth will							
(i) Correct	352	69.4	244	67.8	108	73.5	0.207**
(ii) Incorrect	155	30.6	116	32.2	39	26.5	
K5) After my braces are removed							
(i) Correct	410	80.9	284	78.9	126	85.7	0.076**
(ii) Incorrect	97	19.1	76	21.1	21	14.3	
A1) I think that people who wear braces do not look attractive							
(i) Correct	373	73.6	257	71.4	116	78.9	0.081**
(ii) Incorrect	134	26.4	103	28.6	31	21.1	
A2) I often wait a very long time in the waiting area							
(i) Correct	179	35.3	123	34.2	56	38.1	0.401**
(ii) Incorrect	328	64.7	237	65.8	91	61.9	
A3) Time spent with the orthodontist during my appointment is							
(i) Correct	420	82.8	295	81.9	125	85	0.402**
(ii) Incorrect	87	17.2	65	18.1	22	15	
A4) I am happy with the treatment outcome so far							
(i) Correct	404	79.7	287	79.7	117	79.6	0.974**
(ii) Incorrect	103	20.3	73	20.3	30	20.4	
A5) My orthodontic treatment is expensive							
(i) Correct	378	74.6	257	71.4	121	82.3	0.010**
(ii) Incorrect	129	25.4	103	28.6	26	17.7	
P1) Restrict certain food and alter my dietary habits to maintain braces							
(i) Correct	202	39.8	148	41.1	54	36.7	0.361**
(ii) Incorrect	305	60.2	212	58.9	93	63.3	
P2) Brush and rinse mouth more often so that food does not get stuck in the braces							
(i) Correct	391	77.1	277	76.9	114	77.6	0.883**
(ii) Incorrect	116	22.9	83	23.1	33	22.4	
P3) While undergoing orthodontic treatment, I clean my teeth using _							
(i) Correct	295	58.2	207	57.5	88	59.9	0.624**
(ii) Incorrect	212	41.8	153	42.5	59	40.1	
P4) Brackets/wires often break or dislodge due to my carelessness							
(i) Correct	260	51.3	175	48.6	85	57.8	0.060**
(ii) Incorrect	247	48.7	185	51.4	62	42.2	
P5) Often forget or miss my appointment dates							
(i) Correct	397	78.3	277	76.9	120	81.6	0.245**
(ii) Incorrect	110	21.7	83	23.1	27	18.4	

**Pearson’s Chi Square test

Bold entries highlight the statistically significant value

### Orthodontic treatment related practices

In the orthodontic treatment related practices section of the questionnaire, a vast majority of the respondents provided the correct response for questions P2 and P5, a small majority provided the correct response for questions P3 and P4 and less than half of the respondents provided the correct response for question P1 (Table 2). Results showed that only 39.8% of respondents made an effort to alter dietary habits all of the time while a majority of 60.2% of respondents made an effort to alter dietary habits only some of the time or not at all. (Table 2; P1). More than three-quarters of the respondents (77.1%) carried out regular oral hygiene practices (Table 2; P2) while 58.2% complied with the use of orthodontic toothbrush and cleaning aids (Table 2; P3). The female respondents had significantly better compliance with tooth brushing and use of cleaning aids (*p* = 0.005). Slightly more than half of respondents (51.3%) claimed to have never broken or dislodged their brackets or arch wires (Table 2; P4). 78.3% of respondents claimed to have never forgotten/missed their orthodontic appointments (Table 2; P5). Males were also significantly more likely to forget their appointment dates and miss their appointment with their orthodontist (*p* < 0.001). In general, females scored slightly better than their male counterparts in all their orthodontic treatment related practices except for question P1.

In general, there was a similar distribution of knowledge, orthodontic treatment related attitude and orthodontic treatment related practices between the four ethnic groups. There was a significant difference between ethnic groups with regard to questions K1, K3, A2, A4 and P1. Respondents of Indian ethnicity were significantly less likely to agree that braces helped to correct tooth and jaw irregularities (*p* = 0.019) (Table 5; K1). The Chinese ethnic group had significantly poorer knowledge on the need to comply with the given oral hygiene and dietary instructions (*p* = 0.002) (Table 5; K3). All races felt that they had to wait a long time in the waiting area to see the orthodontist. A significantly higher number of Malays were happy with their orthodontic treatment outcome (*p* = 0.017) (Table 5; A4). The Malays and Chinese were less likely to comply with the practice of restricting certain foods and altering dietary habits during their treatment (*p* = 0.018) (Table 5; P1).

**Table 5:** Responses of study participants based on ethnicity

Questions	Total (N = 507)		Malay		Chinese		Indian		Others		*p* value (< 0.05)
	n	%	n	%	n	%	n	%	n	%	
K1) Braces											
(i) Correct	496	97.8	311	98.4	135	98.5	43	91.5	7	100	0.019**
(ii) Incorrect	11	2.2	5	1.6	2	1.5	4	8.5	0	0	
K2) The duration of undergoing orthodontic treatment is											
(i) Correct	439	86.6	280	88.6	117	85.4	36	76.6	6	85.7	0.150**
(ii) Incorrect	68	13.4	36	11.4	20	14.6	11	23.4	1	14.3	
K3) Instructions on oral hygiene and eating habits											
(i) Correct	497	98	315	99.7	129	94.2	46	97.9	7	100	0.002***
(ii) Incorrect	10	2	1	0.3	8	5.8	1	2.1	0	0	
K4) If the treatment is incomplete, the condition of my teeth will											
(i) Correct	352	69.4	217	68.7	93	67.9	36	76.6	6	85.7	0.522**
(ii) Incorrect	155	30.6	99	31.3	44	32.1	11	23.4	1	14.3	
K5) After my braces are removed											
(i) Correct	410	80.9	251	79.4	117	85.4	35	74.5	7	100	0.179***
(ii) Incorrect	97	19.1	65	20.6	20	14.6	12	25.5	0	0	
A1) I think that people who wear braces do not look attractive											
(i) Correct	373	73.6	232	73.4	96	70.1	40	85.1	5	71.4	0.211***
(ii) Incorrect	134	26.4	84	26.6	41	29.9	7	14.9	2	28.6	
A2) I often wait a very long time in the waiting area											
(i) Correct	179	35.3	127	40.2	38	27.7	12	25.5	2	28.6	0.031**
(ii) Incorrect	328	64.7	189	59.8	99	72.3	35	74.5	5	71.4	
A3) Time spent with the orthodontist during my appointment is											
(i) Correct	420	82.8	265	83.9	117	85.4	33	70.2	5	71.4	0.074***
(ii) Incorrect	87	17.2	51	16.1	20	14.6	14	29.8	2	28.6	
A4) I am happy with the treatment outcome so far											
(i) Correct	404	79.7	265	83.9	98	71.5	98	76.6	5	71.4	0.017***
(ii) Incorrect	103	20.3	51	16.1	39	28.5	339	23.4	2	28.6	
A5) My orthodontic treatment is expensive											
(i) Correct	387	74.6	230	72.8	104	75.9	38	80.9	6	85.7	0.626***
(ii) Incorrect	129	25.4	86	27.2	33	24.1	9	19.1	1	14.3	
P1) Restrict certain food and alter my dietary habits to maintain braces											
(i) Correct	202	39.8	115	36.4	55	40.1	28	59.6	4	57.1	0.018**
(ii) Incorrect	305	60.2	201	63.6	82	59.9	19	40.4	3	42.9	
P2) Brush and rinse mouth more often so that food does not get stuck in the braces											
(i) Correct	391	77.1	239	75.6	106	77.4	40	85.1	6	85.7	0.549***
(ii) Incorrect	116	22.9	77	24.4	31	22.6	7	14.9	1	14.3	
P3) While undergoing orthodontic treatment, I clean my teeth using											
(i) Correct	295	58.2	173	54.7	85	62	31	66	6	85.7	0.126**
(ii) Incorrect	212	41.8	143	45.3	52	38	16	34	1	14.3	
P4) Brackets/wires often break or dislodge due to my carelessness											
(i) Correct	260	51.3	164	51.9	63	46	28	59.6	5	71.4	0.260**
(ii) Incorrect	247	48.7	152	48.1	74	54	19	40.4	2	28.6	
P5) Often forget or miss my appointment dates											
(i) Correct	397	78.3	236	74.7	115	83.9	40	85.1	6	85.7	0.087***
(ii) Incorrect	110	21.7	80	25.3	22	16.1	7	14.9	1	14.3	

**Pearson’s Chi Square test;

***Fisher’s Exact test

Bold entries highlight the statistically significant value

## Discussion

Health-related behaviour (also known as health behaviour) has been shown to directly and indirectly affect an individual’s health either in a positive or negative manner. Knowledge, attitude and practice (KAP) is an essential component of an individual’s health behaviour. Despite its importance, there is a scarcity of health behaviour research especially regarding the KAP of orthodontic patients. Most studies have focused on the “awareness” component of health behaviour. From the perspective of orthodontics, health behaviour may affect clinical outcome due to its influence on attitude and practices related to the patient’s role in successful orthodontic treatment.

The majority of patients in our study were females. This may be due to the generally higher level of awareness related to treatment need as well as demand among females [4, 16]. Overall, females were found to have better levels of KAP and this finding is similar to other KAP studies [13, 14]. In our cohort, statistically significant differences between the two sexes were observed in relation to knowledge on retainer wear, the attractiveness when wearing braces, usage of cleaning aids and remembering as well as attending appointments regularly; with females doing better than males.

The ethnic distribution of the patients in this cohort was reflective of the overall ethnic distribution of the Malaysian population based on the estimates by the Department of Statistics Malaysia [17].

The government or public healthcare system in Malaysia is utilised by all layers of society cutting across all income brackets and this is illustrated in the findings from our study. Interestingly, the vast majority of patients in our cohort were from the B40 household income group. This suggests that patients from the B40 income were also very cognizant of orthodontic treatment needs. Due to the highly subsidized cost of orthodontic treatment in the public health sector, patients from the B40 income group are also able to afford orthodontic treatment. This may be the reason why most respondents felt that orthodontic treatment provided at the public healthcare facilities was inexpensive. However, as there is an extremely high demand, there exists a long wait list. A staggered payment system for the lower income patients has recently been introduced to help ease the burden of payment for some. Suggestion of a tiered payment system where the poor receive a larger subsidized rate than the rich could encourage those from the lower income bracket to seek treatment.

Our study found that overall, most patients had good knowledge of orthodontic treatment however the percentage of positive attitude and orthodontic treatment related practices section were lower. Being aware of the patient’s level of knowledge, attitude and their orthodontic related practices can help the clinician better understand our patients and our role as a clinician. This also enables the orthodontic team to reflect on the areas that we could pay a closer attention to when educating our patients in an orthodontic practice.

In the knowledge section the lowest level of knowledge (69.4%) was regarding the consequences of incomplete orthodontic treatment and this level was much lower than other similar studies which found the level to be somewhere between 79.0% to 90.1% [12–14]. This is an indication that the orthodontic team should include and stress upon the possible risks of incomplete orthodontic treatment during the discussion of the treatment plan with the patients so that the patient is fully aware of consequences and the possibility that dental condition may worsen over time if their orthodontic treatment is not completed.

As for retainer wear, most studies tend to focus on patient compliance, the length of retainer wear and the type of retainer prescribed [18–22]. Not many studies have been carried out on the knowledge of retainer wear among orthodontic patients. A previous study showed that the level of knowledge regarding retainer wear upon completion of treatment can vary from as low as 45.7% to as high as 79.9% [13, 14]. Our study however found that a high percentage 80.9% of patients were aware of the need to wear retainers which shows that we have been successful in communicating the need for a retention phase to the patients. The awareness on the need for retainer wear upon completion of the active orthodontic treatment cannot be stressed enough and is vital in the overall maintenance of successful orthodontic treatment.

Generally, the respondents had a positive attitude towards their orthodontic treatment experience except where the waiting time to see the orthodontist at every appointment was concerned. We found that a large number of patients 64.7% felt that they wait too long to see their attending orthodontist. Previous studies have found that waiting time is the single most important factor contributing to patient satisfaction and on whether the patient is likely to be compliant for the following visits [23, 24]. As orthodontic treatment requires multiple visits, compliance is a very important factor in determining a successful treatment outcome. An efficient system should be implemented to reduce the waiting time for patients. A good way to decrease the waiting time is using a staggered appointment system. Frequent breakages usually lead to emergency appointments resulting in delays that may negatively affect the appointment / waiting time (as well as duration) of other patients that have been given appointments for that particular day. Reducing breakages will allow the orthodontist to better predict the time needed to see each patient and in turn avoid overrunning the appointment time. However, for this to work the orthodontist needs to keep strictly to time and the patient too needs to adhere strictly to their appointed time and minimize breakages; patient education is integral. Interventions such as videos, magazines, health talks, visual art and information by oral health personnel in the waiting area have proven to reduce the waiting stress and to improve the waiting experience [23]. Attitude can be moulded prior to starting the treatment and during the initial visits as well as during the bond up appointment through chair side instructions and handing out the relevant patient information sheets. Studies have shown that patients who have had previous orthodontic treatment [8, 9, 25] and those whose parents have had orthodontic treatment were more likely to have a positive attitude towards orthodontic treatment [26]. Though pain was not assessed in this study, the experience of pain is thought to be an important factor that can influence the patient’s attitude towards orthodontic treatment [7, 27, 28].

Despite patients having good knowledge regarding orthodontic treatment, orthodontic treatment related practices of patients in general were at a much lower level. Oral hygiene practices are a very important part of orthodontic treatment to prevent development of oral diseases during orthodontic treatment [29]. Frequent reinforcement of oral hygiene and motivation not just at the initial stages of orthodontic treatment but throughout the treatment should be carried out as it has been shown to improve patients’ attitude and oral hygiene practices [30, 31]. Some studies show that a leaflet and other promotional materials can also help to improve the patients understanding and oral hygiene practices [32]. The failure to alter dietary habits can directly lead to a high number of breakages. As such, more effort needs to be placed on emphasizing the importance of avoiding hard, sticky and large mouthfuls throughout the treatment duration to reduce breakages and to reduce emergency appointments that in turn may further burden the long waiting time of patients.

In this study only slightly more than half the respondents made use of special cleaning aids. We found a large variation in the usage of special cleaning aids within the existing literature, ranging from 25.3 to 100% [33–35]. Although this is an area of interest, the current study was not designed to look at the reasons behind this poor orthodontic treatment related behaviour. Studies looking into this particular aspect of orthodontic treatment related behaviour are very much needed to better understand the patient’s perspective and to enable remedial measures to be put into place. Good oral hygiene practices and regular fluoride application are important during the fixed appliance stage as fixed appliance can easily trap food and harbour plaque which can lead to patient developing gingivitis, periodontitis, dental caries and halitosis.

As the orthodontist sees their patients frequently and on a regular interval for a number of years, their ability to give good oral health education can influence healthy oral health practice in the patient. Hence, more attention needs to be given to the patients especially after their initial bond-up appointment to encourage patients to use recommended cleaning aids. Periodic reinforcement of brushing instructions and dietary restrictions during the follow-up appointments are important to improve patient’s practices. Giving appropriate and adequate information, repeated motivation and practice to patients has been shown to change the patient’s attitude and practice to a more positive one [36]. A good orthodontist-patient relationship can also improve the patient’s attitude and compliance towards treatment [37, 38].

There were several limitations to our study. The personality traits of patients were not looked at although they could be a contributing factor to their attitude. Reasons for the patient’s poor orthodontic related practices were also not investigated in this study as this was beyond the scope of the study. This study was also focused on those undergoing only fixed appliance therapy, as inclusion of other treatment measures would make the population too heterogenous. We were also unable to do a sub-analysis assessing the relationship between KAP and time to treatment onset. However. this is something we plan to look at during future studies.

## Conclusions

Several interesting features regarding the KAP of patients in relation to their orthodontic treatment were discovered through this study. Even though a large majority of patients were found to have good knowledge but attitude towards orthodontic treatment and orthodontic treatment related practices were not as good. The orthodontics related practice with the lowest score was related to patients not strictly adhering to dietary restrictions during their orthodontic treatment. The level of usage of special cleaning aids and the frequency of breakages were also aspects that need to be improved. The orthodontic team can play a more active role in improving orthodontics related practices among patients through better patient education.

## Data Availability

The datasets used and/or analysed during the current study are available from the corresponding author upon reasonable request.

## Abbreviations

KAP: Knowledge, attitude and practice
SD: Standard deviation
